# Association of growth hormone and insulin-like growth factor I genotype with body weight, dominance of body weight, and mRNA expression in Korat slow-growing chickens

**DOI:** 10.5713/ab.20.0729

**Published:** 2021-03-10

**Authors:** Panpradub Sinpru, Rujjira Bunnom, Chotima Poompramun, Pramin Kaewsatuan, Sirangkun Sornsan, Satoshi Kubota, Wittawat Molee, Amonrat Molee

**Affiliations:** 1 School of Animal Technology and Innovation, Institute of Agricultural Technology, Suranaree University of Technology, Nakhon Ratchasima, 30000, Thailand

**Keywords:** Body Weight, Growth Hormone, Heterosis, Insulin-like Growth Factor-I, Slow-growing Chicken

## Abstract

**Objective:**

Growth hormone (*GH*) and insulin-like growth factor I (*IGF-I*) play a critical role in animal growth rates. We aimed to investigate the effect of *GH* and *IGF-I* genotypes on body weight (BW), dominance, and gene expression in slow-growing chickens at different ages.

**Methods:**

A total of 613 Korat chickens (KRs) were bred and divided into three groups by genotype – A1A1, A1A3, and A3A3 for *GH* and AA, AC, and CC for *IGF-I*. Chickens were weighed every two weeks, and liver and breast muscle tissues were collected at 10 weeks of age. Genetic parameters of KRs were estimated using ASReml software. The *GH* and *IGF-I* mRNA levels were measured by quantitative polymerase chain reaction. Significant differences between traits were analyzed using the generalized linear model.

**Results:**

A significant effect of *GH* genotypes on BW was found at most ages, and the A1A1 genotype had the highest value of BW. Compared with the A3A3 genotype, the A1A1 and A1A3 genotypes showed a higher dominance effect at 0 and 2 weeks, and genotype A1A1 had the highest value of dominance at 8 weeks of age. A difference in *GH* mRNA levels between genotypes was detected in breast muscle at 6 weeks and in the liver tissue at 2 weeks. In the case of *IGF-I* gene, the AA genotype had the highest BW at the beginning of life. Significant differences in BW dominance were found at 2 weeks. However, *IGF-I* mRNA levels were not different among genotypes in both breast muscles and liver tissues.

**Conclusion:**

Our results revealed that *GH* and *IGF-I* influence growth, but may not be involved in heterosis. *GH* can be used as a marker gene in selection programs for growth because the homozygous genotype (A1A1) had the highest BW at all ages. The *IGF-I* is not a useful marker gene for selection programs.

## INTRODUCTION

Slow-growing chickens are an alternative to broiler chickens that can be kept by smallholder farmers, particularly in developing countries because of their outstanding meat flavor and because they can be raised in a non-intensive system that can reduce production costs [1]. The slow-growing chickens’ improved performance will help developing countries to achieve at least three of the sustainable development goals (SDGs) set by the United Nations: no poverty, zero hunger, and good health [2]. Korat chicken (KR) is a crossbred chicken; its sire and dam are Leung Hang Khao (LK) and a Suranaree University of Technology (SUT) line, respectively. This chicken has been used as a national tool to achieve the first three SDGs, promote smallholder farmers, and improve their income. KR is defined as a slow-growing chicken, as its average daily weight gain and feed conversion ratio is 19.8 to 21.0 g/d and 2.2 to 2.3, respectively [3]. Therefore, it is necessary to improve the growth performance of KRs to expand the sustainability of smallholder farmers in the country.

Growth performance is affected by heterosis, which has been exploited in the chicken breeding industry [4]. Previous studies have investigated the relationship between gene expression and heterosis in chickens. Wang et al [5] have reported differential insulin-like growth factor-I (*IGF-I*) gene expression between parental and F1 hybrid chickens and its relationship to heterosis of meat traits. Sun et al [6] have investigated differences in gene expression in the liver between chicken hybrids and their parents in 4×4 diallelic crosses. Their results indicated that patterns of gene expression in hybrids differed significantly from those of their parents. A genome-wide association technique has been used to investigate the genetic basis of heterosis for a fatness trait in two F2 populations of meat-type broilers [7]. This study demonstrated that heterosis is affected by epistasis. The results of these studies showed a strong connection between heterotic effects and gene function. However, the association between heterosis and specific loci needs to be investigated to identify specific marker genes for improving mating selection [8].

A number of genes are involved in growth traits; however, growth hormone (GH) and IGF-I are the major hormones required to support normal growth [9,10]. The GH is involved in a wide variety of physiological functions, such as growth, body composition, egg production, aging, and reproduction [11], and it plays a critical role in both growth and metabolism rates [12,13]. The IGF-I is a hormone that is structurally related to insulin and has multifunctional metabolic and anabolic properties [14]. It is a crucial hormone for normal growth, and for bone and fat tissue development in chickens [15–18].

The *GH* and *IGF-I* are expressed in many tissues, such as the pituitary, heart, liver, and muscle [19]. However, in the liver, GH functions in the intermediate pathway between the pituitary gland and one of its target tissues, the muscle [20]. Rastegar et al [21] have reported that GH in the liver is likely to control the synthesis of a whole set of proteins by regulating their gene expression. The GH stimulates IGF-I synthesis in the liver and regulates the paracrine production of IGF-I in many other tissues [22]. Moreover, *GH* and *IGF-I* are promising candidate marker genes for improving the growth rate of chickens [13,23]. Different *GH* and *IGF-I* genotypes are predicted to induce different levels of gene expression and different effects on body weight (BW) [16,24,25]. Additionally, BW, age, and tissues have induced changes in the expression profiles of the *GH* and *IGF-I* genes, suggesting that altering the regulatory mechanisms of these genes may improve growth traits in chickens [23]. The objectives of this study were to investigate the effect of *GH* and *IGF-I* genotypes on BW, dominance, and their expression levels in the liver and breast muscle at different ages of KRs. The results will be useful for mating selection programs to improve the growth performance of slow-growing chickens.

## MATERIALS AND METHODS

### Ethics statement

All experiments were conducted at the Poultry Research Unit of the University Farm, Suranaree University of Technology. All procedures used in the present study were approved by the Ethics Committee on Animal Use of Suranaree University of Technology, Nakhon Ratchasima, Thailand (document ID U1-02631-2559).

### Animals and experimental design

To study the effect of genotypes on growth performance of KRs, the *GH* and *IGF-I* genotypes of 142 LK male and 259 SUT female parent chickens were determined. A total of 613 mixed-sex KRs were produced by mating pairs of parents based on their genotype at the Poultry Research Unit of the University Farm, Suranaree University of Technology. The number of sires, dams, and KRs with each genotype of both genes is presented in Table 1. A completely randomized experimental design was applied, and each locus was divided into three groups by genotype—A1A1, A1A3, and A3A3 for *GH* and AA, AC, and CC for *IGF-I*—with four replications. The number of chickens per replication was A1A1 (n = 33), A1A3 (n = 24 to 25), A3A3 (n = 29 to 30) for *GH* gene, and AA (n = 8 to 9), AC (n = 28 to 29), CC (n = 29 to 30) for *IGF-I* gene. KRs were raised following SUT farm guidelines in open-sided housing, and had access to feed and water *ad libitum*. The feeding program was designed for three different periods: 0 to 4, 5 to 8, and 9 to 10 weeks. The basal diet at the initial, second, and final periods contained 21%, 19%, and 17% protein, respectively.

### Data and samples collection

Individual BW of KRs was recorded at the age of 0, 2, 4, 6, 8, and 10 weeks. Blood samples of LK and SUT chickens were collected to extract genomic DNA and genotype the *IGF-I* and *GH* genes. When the KRs reached 2, 4, 6, 8, and 10 weeks of age, one chicken from each replication were randomly selected and sacrificed (4 chickens per time point) for gene expression study. The chickens were stunned with chloroform after weighing and sacrificed by cutting the carotid artery and jugular vein in their neck. The skin over the abdomen was cut and removed and the liver and breast muscle tissues were collected, immediately frozen in liquid nitrogen, and stored at −80°C until they were used for RNA analysis.

### Heterosis of body weight

Outlier and normal distribution were tested before use in statistical analysis, and mean, standard deviation, minimum, and maximum were determined. The heterosis of BW at each week was calculated using least square mean (Table 2), where sex and hatch date were a fixed effect. The following formula of heterosis calculation was used:

$$H\%=\frac{{LSM}_{KR}-\frac{\left({LSM}_{LK}+{LSM}_{SUT}\right)}{2}}{\frac{\left({LSM}_{LK}+{LSM}_{SUT}\right)}{2}}\times 100$$

Where *LSM_KR_* = the least square mean of BW of KR; *LSM_LK_* = the least square mean of BW of sire line (LK); *LSM_SUT_* = the least square mean of BW of dam line (SUT).

### Dominance estimation

Dominance of BW at 0, 2, 4, 6, 8, and 10 weeks of age was estimated. There were 9,079 BW and pedigree data records from the 1st to the 5th generation those compose with 5,800, 9,640, and 9,868 of LK, SUT, and KR respectively. The animal model for dominance is shown below, and restricted maximum likelihood was used to estimate the effect and genetic parameters, using ASReml software [26]. Genetic parameters, variance component, heritability, and the ratio between variance dominance and total variance are shown in Table 3.

$$\begin{array}{l} y=X\beta +Za+Wd+ɛ\\ V\left[\begin{array}{c} a\\ d\\ ɛ \end{array}\right]=\left[\begin{array}{ccc} A\sigma_{a}^{2} & 0 & 0\\ 0 & D\sigma_{d}^{2} & 0\\ 0 & 0 & I\sigma_{ɛ}^{2} \end{array}\right] \end{array}$$

Where y is the vector of the KR BW at 0, 2, 4, 6, 8, and 10 weeks of age, *β* is the vector of the fixed effect, which includes hatch date and sex, *a* is the vector of the additive effect, *d* is the vector of dominance, and *ɛ* is the vector of unidentified factors. *X*, *Z*, *W* are matrices of fixed effect, additive effect, and dominance, respectively, and *A*, *D*, *I* are matrices of relationship in a population, mating relationship, and identity matrix, respectively. $\sigma_{a}^{2},\sigma_{d}^{2},\sigma_{ɛ}^{2}$ are additive, dominance, and error variances, respectively.

### Genotyping

The genomic DNA of the LK and SUT chickens was isolated from whole blood using a Blood/Tissue DNA Mini Kit following the manufacturer’s instructions (Geneaid Biotech Ltd., New Taipei, Taiwan). Polymerase chain reaction (PCR)-restriction fragment length polymorphism (RFLP) was used to identify *GH* and *IGF-I* genotypes. Forward and reverse primers, enzymes, and annealing temperatures for PCR-RFLP of *GH* and *IGF-I* gene are shown in Table 4. Regarding the *IGF-I* gene, the primer sets were used for amplification of an 813-bp fragment containing a single nucleotide polymorphism (SNP) at the promoter region and 5′ untranslated region of *IGF-I* [27]. The genotype of intron I of *GH* was determined following a method described by Thakur et al [28].

### Gene expression analysis

Total RNA was isolated from liver and breast muscle at each age using TRIzol Reagent (Invitrogen; Thermo Fisher Scientific, Waltham, MA, USA). RNA quality was assessed using a spectrophotometer (Nanodrop 2000, Thermo Fisher Scientific, USA). First stand cDNAs were synthesized using a Transcriptor First Strand cDNA Synthesis Kit (Applied Biosystems, Foster City, CA, USA). cDNA (2 μL) was mixed with 6 μL of deionized water, 10 μL of SYBR Green I Master (Applied Biosystems, USA), and 1 μL of each forward and reverse primer. The primers were designed using NCBI Primer BLAST ( https://www.ncbi.nlm.nih.gov/tools/primer-blast/). Real-time PCR was performed using the LightCycler 480 Real-Time PCR System (Roche, Germany). *GH* and *IGF-I* expression levels in breast muscle were normalized to the expression level of ribosomal protein L13 (*RPL13*) and glyceraldehyde-3-phosphate dehydrogenase (*GAPDH*), respectively, whereas, in the liver, both target genes were normalized to the level of 18S ribosomal RNA (*18S*).

### Statistical analysis

Correlation between BW and dominance was analyzed by Pearson’s Correlation. Significant differences in BW, dominance, and expression levels between genotypes were analyzed using the generalized linear model. Sex was fixed, and Tukey’s range test was used to compare means. Significant difference was accepted at p≤0.05. SPSS 18.0 software package (SPSS Inc., Chicago, IL, USA) was used for all statistical procedures.

## RESULTS AND DISCUSSION

### Heterosis and characteristics of body weight of Korat chickens

Table 2 presents percent heterosis, BW characteristics, and correlation between BW and dominance. KRs are slow-growing chickens that need around 10 weeks for their BW to reach 1.2 kg, which is the market size. The effect of heterosis on the chickens’ BW from hatching to 10 weeks of ages was positive and ranged from 12% to 28% (% heterosis at 2 weeks was not calculated because the parents were unidentified). These results suggest that the heterosis effect was small when the chickens hatched and it increased with age. The results were consistent with reports by Taha and AbdEl-Ghany [29] and Nwenya et al [30], who found that F1 crossbred chickens showed the lowest percentage of heterosis at 0 weeks and that it increased with age, ranging from 9% to 14% and 10% to 17% in male Egyptian chicken and Naked Neck× Frizzled Feather, respectively. A possible reason for the influence of age on the heterosis effects is that the non-additive gene effects (dominance, overdominance and epistasis) affect these traits more when the environmental conditions are less favorable [31]. These results confirmed the success of using crossbreeding for exploiting heterosis in the KR population.

Assessing the correlation between BW and dominance was performed first because heterosis influenced BW, therefore, we hypothesized that BW must have a significant positive correlation with dominance. The results of this study were consistent with this hypothesis, i.e., a significant positive correlation between BW and dominance was detected at all ages, except for 0 weeks (p<0.05). Moreover, the correlation coefficient continually increased with the age of chickens from 0.442 at 4 weeks of age to 0.830 and 0.710 at 8 and 10 weeks of age, respectively. These results indicated that the dominance effect plays a role in the BW trait, and that this effect increases with age. This finding is consistent with a study by Tang et al [32], who found that the dominance effect associated with BW at 8, 10, and 13 weeks, i.e., it increases with age. This suggests that the dominance effect does not exist at hatching because the maternal genetic and maternal permanent environmental effects have more influence on BW, however it begins to gradually increase with age. It could be that several genes control BW at a later stage of growth and that there is an increase in the combination between different favorable alleles [33].

### Genetic parameters

The variance component and heritability of BW from 0 to 10 weeks of age are reported in Table 3. The lowest additive variance was for hatching weight, but it increased with age. The range of the heritability of BW traits varied from 0.53 to 1.00. The ratio of dominance and total variance was 0.00 at 0–4 weeks and 0.18, 0.04, and 0.17 at 6, 8, and 10 weeks, respectively.

The high heritability of the BW trait at hatching is consistent with a report by Ebrahimi et al [31], who found that heritability of the BW trait at hatching of Japanese quails was high, because at hatching the BW of the animals was barely influenced by the environmental effect, however this relationship changed with age. A low ratio of dominant genetics to total phenotypic variance, 6%, 8%, and 1%, has been reported for BW at 0, 8, and 12 weeks of age, respectively, in Mazandaran fowls, a dual-purpose chicken [34]. However, a relatively higher proportion of dominance effect, 18%, 4%, and 17% at 6, 8, and 10 weeks of age, respectively, was detected in our study, probably because KR is a crossbred chicken of LK and SUT, designed to enhance growth performance by hybrid vigor. The results showed that the BW trait of KRs was influenced by both additive and dominant genetic effects, indicating that selection for higher BW could be applied to improve growth of KR.

### Effect of growth hormone genotype on body weight, dominance, and gene expression

An association study between the *GH* genotype and BW, dominance, and *GH* expression was conducted (Table 5). A significant genotype effect on BW and dominance was detected at all of ages (p<0.05) and at 0, 2, and 8 weeks of age (p<0.05), respectively. For dominance, the A1A1 and A1A3 genotypes had the largest effect at 0 and 2 weeks of age compared with the A3A3 genotype, and the A1A1 genotype had the largest effect at 8 weeks of age compared with both the A1A3 and A3A3 genotypes. Moreover, a significant difference between *GH* genotypes and its expression level in liver or breast was only found at 2 and 6 weeks, respectively.

Chickens with the A1A1 genotype showed higher BW than the other genotypes. However, the dominance effect of chickens with the A1A1 genotype was not significant at 0 and 2 weeks compared with the chickens with the A1A3 genotype, whereas the A1A1 genotype had the largest effect at 8 weeks. This result is consistent with the findings of Anh et al [13], who reported that there was no significant association between the *GH* gene at intron 3 and BW in Thai boilers when comparing between a heterozygous genotype and a homozygous genotype, in which complete dominance of allele G over A of *GH* gene was observed. Although the dominance effect was found to be significantly associated with BW at all ages in our study (Table 2), it appears that the *GH* locus did not exhibit an overdominance effect in chickens with the A1A3 genotype. The dominance effect contributes to the hybrids based on the sum of the effects of heterozygous loci across the whole genome. A high degree of dominance effect depends on superior alleles originating from divergent parents, resulting in better performance than the parents [35]. Therefore, the *GH* gene influences growth, but the study of a single locus may not be sufficient to consider the contribution of the overall effect of dominance on BW.

The expression levels in both breast and liver did not correlate with BW and dominance, as non-significant differences between *GH* expression and genotype were found in most ages. Although the association between *GH* gene polymorphism and growth traits has been studied in chickens [13, 24], the relationships of the *GH* genotypes and gene expression profiles with BW of chickens are not well understood. Studies on *GH* gene product may explain this inconsistency, and thus further investigations are required.

### Association between insulin-like growth factor I and body weight, dominance, and gene expression

The effects of genotype on BW, dominance, and *IGF-I* mRNA levels are presented in Table 6. Significant effects of *IGF-I* genotypes on BW were detected at 0, 2, and 4 weeks of age (p<0.05) and chickens with the AA genotype showed the highest BW. Significant effects on dominance were only observed at 2 weeks of age (p<0.05). However, there were no significant differences in *IGF-I* mRNA levels between genotypes in either breast muscle or liver tissues.

The significant effects of the *IGF-I* genotypes on BW at 0, 2, and 4 weeks of age occurred when the growth rate was accelerating. However, the effect of the heterozygous genotype was not higher than that of the homozygous genotypes, of which the homozygous AA genotype had the largest effect at 2 and 4 weeks of age. These results are similar to the results of Anh et al [13], who studied an SNP within the same region and reported that crossbred chickens with the homozygous AA genotype showed higher BW at 2 weeks than the chicken with the heterozygous AC genotype. Although the dominance effect was found to be significantly associated with BW at all ages in our study (Table 2), Su et al [36] and Zhang et al [37] have reported that the heterotic effect and the dominance effect of this trait are controlled by other genes. These findings suggest that *IGF-1* influences BW in crossbred chickens at young ages [18], however it may not be involved in heterosis.

Furthermore, comparison of *IGF-I* gene expression levels among the three genotypes (AA, AC, and CC) did not agree with the BW results. No significant differences were detected in *IGF-I* mRNA levels among the three genotypes in both breast muscle and liver tissues. Moe et al [27], who studied the promoter and 5′-untranslated regions of the *IGF-I* gene, also found that *IGF-I* mRNA expression in the liver of laying chickens was not different among the AA, AC, and CC genotypes, though there was a difference in BW. However, our results were inconsistent with the study of Amills et al [38], who suggested that polymorphism of the *IGF-I* gene led to differences in gene expression. In swine, the *IGF-I* microsatellite may be located at the 5′ region of the gene close to the promoter region and linked to some other loci in the same chromosome region, which is responsible for the effect on growth traits [39]. Perhaps the difference in BW among *IGF-I* genotypes in this study is due to a linkage with other loci in the same chromosome region. This may explain the lack of significant difference in gene expression among the genotypes.

In summary, the data presented in this study show that *GH* and *IGF-I* influence BW, but they may not be involved in heterosis. Moreover, the results at the molecular level clearly indicate that heterosis is not associated with the *GH* and *IGF-I* genotypes. Growth is a complicated trait and many genes control chicken growth at different stages. Our study indicates that *GH* can be used as a gene marker because there was a strong effect of the homozygous A1A1 genotype on BW. However, *IGF-I* is not a useful gene marker for KR selection programs.

## Figures and Tables

**Table 1:** The number of Suranaree University of Technology and Leung Hang Khao chickens for designing mating plans to produce Korat chickens in each gene group

Gene	Genotype of SUT (Dam)	No. of SUT	Genotype of LK (Sire)	No. of LK	Genotype of KR	No. of KR
*GH*	A1A1	35	A1A1	13	A1A1	132
	A1A1	22	A3A3	20	A1A3	97
	A3A3	34	A3A3	28	A3A3	117
*IGF-I*	AA	63	AC	18	AA	35
	AA	80	CC	42	AC	114
	CC	25	CC	21	CC	118
	Total	259	Total	142	Total	613

SUT, Suranaree University of Technology; LK, Leung Hang Khao chickens; KR, Korat chickens; *GH*, growth hormone; *IGF-I*, insulin-like growth factor I.

**Table 2:** Body weight (g) characteristics of Korat chickens

Age (wk)	Number of samples	Mean	CV (%)	Min	Max	Heterosis (%)	Dominance	Correlation between BW and dominance
0	611	43.05	10.5	31.55	57.50	12	−7.10×10^−7^	0.374 (0.072)
2	608	165.67	16.1	105.00	390.00	NA	0.53×10^−8^	0.544 (0.006)
4	582	394.71	16.0	270.00	610.00	25	6.90×10^−7^	0.442 (0.031)
6	552	715.45	16.0	420.00	1,100.00	25	3.37	0.574 (0.003)
8	515	1,009.20	18.4	610.00	1,580.00	27	0.20	0.830 (0.000)
10	471	1,289.90	18.7	820.00	2,000.00	28	−2.11	0.710 (0.000)

BW, body weight; CV, coefficient of variation; NA, parents unidentified.

**Table 3:** Genetic parameters of body weight gain in Korat chickens

Genetic	Age (wk)					

	0 week	2 weeks	4 weeks	6 weeks	8 weeks	10 weeks
$\sigma_{a}^{2}$	13.49	165.71	3,721.66	14,250.04	14,030.04	15,629.51
$\sigma_{d}^{2}$	0.00	0.00	0.00	3,314.70	1,118.85	6,021.84
$\sigma_{e}^{2}$	0.00	344.25	702.17	712.57	10,033.81	14,661.96
$\sigma_{p}^{2}$	13.49	509.96	4,423.83	18,277.31	25,182.69	36,313.32
*h* ^2^	1.00	0.32	0.84	0.78	0.56	0.43
Standard error	0.00	0.10	0.03	0.03	0.03	0.03
$\frac{\sigma_{d}^{2}}{\sigma_{p}^{2}}$	0.00	0.00	0.00	0.18	0.04	0.17

**Table 4:** Primer sequences and their applications

Genes	5′ sequence 3′ forward/reverse primers	Annealing temperatures (°C)	PCR product size (bp)	Accession no.	Technique	Reference
*GH*	5′-ATCCCCAGGCAAACATCCTC-3′ 5′-CCTCGACATCCAGCTCACAT-3′	60	776	EF452679.1	PCR-RFLP (Msp-I)	Thakur et al [28]
*IGF-I*	5′-CATTGCGCAGGCTCTATCTG-3′ 5′-TCAAGAGAAGCCCTTCAAGC-3′	61.4	813	M74170	PCR-RFLP (Hinf-I)	Moe et al [27]
*GH*	5′-AGTGGCCACAAATCAGCAAG-3′ 5′-TCCGGACATTCTTTCCAGTCT-3′	62	142	S68571.1	Real-time PCR	In this study
*IGF-I*	5′-TATGGATCCAGCAGTAGACGC-3′ 5′-CTCCTCAGGTCACAACTCTGG-3′	60	77	NM_001004384.2	Real-time PCR	In this study
*GAPDH*	5′-GGTGGCCATCAATGATCCCT-3′ 5′-CCGTTCTCAGCCTTGACAGT-3′	58	105	NM_204305.1	Real-time PCR	In this study
*RPL13*	5′-AGGTGCCCGACTGTCAGATA-3′ 5′-GAGCGATACTCCTTCAGCCG -3′	62	188	NM_204999.1	Real-time PCR	In this study
*18s*	5′-GTTCAGCCACCCGAGATTGA-3′ 5′-CCCATCACGAATGGGGTTCA-3′	60	145	XR_003078044.1	Real-time PCR	In this study

PCR-RFLP, polymerase chain reaction-restriction fragment length polymorphism; *GH*, growth hormone; *IGF-I*, insulin-like growth factor I; *GAPDH*, glyceraldehyde-3-phosphate dehydrogenase; *RPL13*, ribosomal protein L13; *18s*, 18S ribosomal RNA.

**Table 5:** Least square means and standard errors of body weight, heterosis, and dominance effect in each growth hormone genotype group of Korat chickens

Traits	Age (wk)	Least square mean±SE of each genotype of *GH* gene (number of chicken)		

		A1A1	A1A3	A3A3
BW (gram)	0	45.06±0.41^a^ (131)	43.38±0.44^b^(97)	41.71±0.42^c^ (116)
	2	203.73±2.51^a^ (130)	171.53±2.63^b^(97)	159.81±2.53^c^ (116)
	4	467.75±5.79^a^ (124)	397.28±6.09^b^(93)	377.69±5.74^c^ (112)
	6	754.78±20.20^a^ (120)	682.52±22.25^b^(86)	673.51±21.71^b^(108)
	8	1,166.00±12.97^a^ (112)	1,004.00±15.46^b^(78)	1,013.00±13.46^b^(101)
	10	1,507.00±54.92^a^ (102)	1,266.00±57.17^b^(72)	1,217.00±56.59^b^(93)
Dominance	0	1.34×10^−6^±0.00^a^ (131)	1.53×10^−6^±0.00^a^ (97)	−8.53×10^−6^±0.00^b^(116)
	2	2.20×10^−6^±0.00^a^ (130)	1.78×10^−6^±0.00^a^ (97)	−1.00×10^−6^±0.00^b^(116)
	4	1.97×10^−6^±0.00 (124)	1.17×10^−6^±0.00 (93)	−8.32×10^−8^±0.00 (112)
	6	3.21±2.09 (120)	5.87±2.34 (86)	6.57±2.20 (108)
	8	2.72±0.61^a^ (112)	−0.62±0.68^b^(78)	0.77±0.64^b^(101)
	10	4.84±2.33 (102)	0.82±2.61 (72)	2.76±2.45 (93)
Normalize	2	1.08±0.03 (4)	1.08±0.02 (4)	1.06±0.03 (4)
Expression	4	1.57±0.22 (4)	1.93±0.16 (4)	1.68±0.22 (4)
Threshold	6	2.03±0.16^ab^ (4)	2.35±0.15^a^ (4)	1.38±0.18^b^(4)
In breast	8	1.63±0.18 (4)	2.23±0.18 (4)	2.05±0.18 (4)
Muscle	10	2.56±0.30 (4)	1.64±0.35 (4)	1.70±0.28 (4)
Normalize	2	1.05±0.01^a^ (4)	1.05±0.01^a^ (4)	1.02±0.01^b^(4)
Expression	4	1.16±0.03 (4)	1.12±0.02 (4)	1.10±0.03 (4)
Threshold	6	1.11±0.03 (4)	1.13±0.03 (4)	1.10±0.03 (4)
In the liver	8	1.10±0.03 (4)	1.13±0.03 (4)	1.14±0.03 (4)
	10	1.08±0.05 (4)	1.20±0.06 (4)	1.13±0.05 (4)

SE, standard error; GH, growth hormone; BW, body weight.

Results were averaged from six individual.

a–c Values with different superscripted letters indicate significant difference of means within the same rows (Tukey’s HSD, p<0.05).

**Table 6:** Least square means and standard errors of body weight, heterosis, and dominance effect in each insulin-like growth factor I genotype group of Korat chickens

Parameter	Age (wk)	Least square mean±SE of each Genotype of *IGF-I* gene (number of chicken)		

		AA	AC	CC
BW (gram)	0	48.83±0.78^a^ (35)	41.96±0.47^b^ (114)	43.07±0.45^ab^ (118)
	2	184.90±2.84^a^ (35)	159.74±1.71^b^ (112)	155.36±1.71^b^ (118)
	4	416.97±8.17^a^ (31)	391.55±5.27^b^ (108)	380.94±5.21^b^ (114)
	6	681.98±27.39 (25)	685.92±22.61 (103)	666.67±21.56 (110)
	8	913.60±38.88 (21)	973.36±26.27 (99)	929.48±26.39 (104)
	10	1,223.00±47.34 (14)	1,225.00±24.79 (94)	1,214.00±25.99 (96)
Dominance	0	2.93×10^−6^±0.00 (35)	−2.75×10^−6^±0.00 (114)	7.48×10^−7^±0.00 (118)
	2	1.99×10^−6^±0.00^a^ (35)	1.78×10^−6^±0.00^a^ (112)	−1.00×10^−6^±0.00^b^ (118)
	4	−7.10×10^−7^±0.00 (31)	1.64×10^−6^±0.00 (108)	3.25×10^−7^±0.00 (114)
	6	−1.94±3.16 (25)	5.49±1.85 (103)	1.42±1.80 (110)
	8	−1.63±1.08 (21)	1.25±0.63 (99)	−0.89±0.61 (104)
	10	−9.74±4.05 (14)	−4.19±2.37 (94)	−6.93±2.31 (96)
Normalize	2	1.05±0.01 (4)	1.05±0.01 (4)	1.04±0.01 (4)
Expression	4	1.06±0.02 (4)	1.05±0.02 (4)	1.07±0.02 (4)
Threshold	6	1.05±0.01 (4)	1.04±0.01 (4)	1.04±0.01 (4)
In Breast	8	1.03±0.02 (4)	1.05±0.02 (4)	1.03±0.02 (4)
Muscle	10	1.05±0.01 (4)	1.06±0.01 (4)	1.06±0.01 (4)
Normalize	2	1.07±0.02 (4)	1.12±0.02 (4)	1.11±0.02 (4)
Expression	4	1.09±0.03 (4)	1.08±0.02 (4)	1.06±0.03 (4)
Threshold	6	1.08±0.03 (4)	1.19±0.03 (4)	1.08±0.03 (4)
In The Liver	8	1.05±0.07 (4)	1.12±0.07 (4)	1.09±0.07 (4)
	10	1.11±0.02 (4)	1.09±0.02 (4)	1.12±0.02 (4)

SE, standard error; IGF-I, insulin-like growth factor I; BW, body weight.

a,b Values with different superscripted letters indicate significant difference of means within the same rows (Tukey’s HSD, p<0.05).

## References

[1] Sarica M, Yamak US, Turhan S, Boz MA, Saricaoğlu FT, Altop A (2014). Comparing slow-growing chickens produced by two- and three-way crossings with commercial genotypes. 2. Carcass quality and blood parameters. Eur Poult Sci.

[2] UN (2018). The sustainable development goals report.

[3] Kubota S, Vandee A, Keawnakient P, Molee W, Yongsawatdikul J, Molee A (2019). Effects of the *MC4R*, *CAPN1*, and *ADSL* genes on body weight and purine content in slow-growing chickens. Poult Sci.

[4] Williams SM, Price SE, Siegel PB (2002). Heterosis of growth and reproductive traits in fowl. Poult Sci.

[5] Wang W, Ouyang K, Ouyang J, Li H, Lin S, Sun H (2004). Polymorphism of insulin-like growth factor I gene in six chicken breeds and its relationship with growth traits. Asian-Australas J Anim Sci.

[6] Sun DX, Wang D, Yu Y, Zhang Y (2005). Cloning and characterization of liver cDNAs that are differentially expressed between chicken hybrids and their parents. Asian-Australas J Anim Sci.

[7] Abasht B, Lamont S (2007). Genome-wide association analysis reveals cryptic alleles as an important factor in heterosis for fatness in chicken F2 population. Anim Genet.

[8] Kaeppler S (2012). Heterosis: Many genes, many mechanisms—End the search for an undiscovered unifying theory. Int Sch Res Notices.

[9] McCann-Levorse LM, Radecki SV, Donoghue DJ, Malamed S, Foster DN, Scanes CG (1993). Ontogeny of pituitary hormone and growth hormone mRNA in the chicken. Proc Soc Exp Biol Med.

[10] Kita K, Nagao K, Okumura J (2005). Nutritional and tissue specificity of IGF-I and IGFBP-2 gene expression in growing chickens - A Review. Asian-Australas J Anim Sci.

[11] Su YJ, Shu JT, Zhang M (2014). Association of chicken growth hormone polymorphisms with egg production. Genet Mol Res.

[12] Vasilatos-Younken R, Zhou Y, Wang X (2000). Altered chicken thyroid hormone metabolism with chronic GH enhancement *in vivo*: consequences for skeletal muscle growth. J Endocrinol.

[13] Anh NTL, Kunhareang S, Duangjinda M (2015). Association of chicken growth hormones and insulin-like growth factor gene polymorphisms with growth performance and carcass traits in Thai broilers. Asian-Australas J Anim Sci.

[14] McMurtry JP, Francis GL, Upton Z (1997). Insulin-like growth factors in poultry. Domest Anim Endocrinol.

[15] Florini JR, Ewton DZ, Coolican SA (1996). Growth hormone and the insulin-like growth factor system in myogenesis. Endocr Rev.

[16] Zhou H, Mitchell AD, McMurtry JP, Ashwell CM, Lamont SJ (2005). Insulin-like growth factor-I gene polymorphism associations with growth, body composition, skeleton integrity, and metabolic traits in chickens. Poult Sci.

[17] Scanes CG (2009). Perspectives on the endocrinology of poultry growth and metabolism. Gen Comp Endocrinol.

[18] Boschiero C, Jorge EC, Ninov K (2013). Association of IGF1 and KDM5A polymorphisms with performance, fatness and carcass traits in chickens. J Appl Genet.

[19] Wu X, Yan MJ, Lian SY, Liu XT, Li A (2014). GH gene polymorphisms and expression associated with egg laying in muscovy ducks (*Cairina moschata*). Hereditas.

[20] Soendergaard C, Young JA, Kopchick JJ (2017). Growth hormone resistancee—Special focus on inflammatory bowel disease. Int J Mol Sci.

[21] Rastegar M, Lemaigre FP, Rousseau GG (2000). Control of gene expression by growth hormone in liver: key role of a network of transcription factors. Mol Cell Endocrinol.

[22] Laron Z (2001). Insulin-like growth factor 1 (IGF-1): a growth hormone. Mol Pathol.

[23] Jia J, Ahmed I, Liu L (2018). Selection for growth rate and body size have altered the expression profiles of somatotropic axis genes in chickens. PLoS One.

[24] Nie Q, Sun B, Zhang D (2005). High diversity of the chicken growth hormone gene and effects on growth and carcass traits. J Hered.

[25] Pandey NK, Singh RP, Saxena VK (2013). Effect of *IGF1* gene polymorphism and expression levels on growth factors in Indian colored broilers. Livest Sci.

[26] Gilmour AR, Gogel BJ, Cullis BR, Thompson R (2009). ASReml User Guide Release 3.0.

[27] Moe HH, Shimogiri T, Kawabe K (2009). Genotypic frequency in Asian native chicken populations and gene expression using insulin-like growth factor 1 (*IGF1*) gene promoter polymorphism. J Poult Sci.

[28] Thakur MS, Parmar SNS, Tolenkhomba TC (2006). Growth hormone gene polymorphism in Kadaknath breed of poultry. Indian J Biotechnol.

[29] Taha AE, AbdEl-Ghany FA (2013). Improving production traits for El-salam and Mandarah chicken strains by crossing II-Estimation of crossbreeding effects for growth production traits. Int J Nutr Food Eng.

[30] Nwenya JMI, Nwakpu EP, Nwose RN, Ogbuagu KP (2017). Performance and heterosis of indigenous chicken crossbreed (Naked Neck × Frizzled Feather) in the humid tropics. J Poult Res.

[31] Ebrahimi K, Dashab GR, Faraji-Arough H, Rokouei M (2019). Estimation of additive and non-additive genetic variances of body weight in crossbreed populations of the Japanese quail. Poult Sci.

[32] Tang S, Sun D, Ou J, Zhang Y, Xu G, Zhang Y (2010). Evaluation of the *IGFs* (*IGF1* and *IGF2*) genes as candidates for growth, body measurement, carcass, and reproduction traits in Beijing You and Silkie chickens. Anim Biotechnol.

[33] Sun LY, Al-Regaiey K, Masternak MM, Wang J, Bartke A (2005). Local expression of GH and IGF-1 in the hippocampus of GH-deficient long-lived mice. Neurobiol Aging.

[34] Jasouria M, Zamania P, Alijanib S (2017). Dominance genetic and maternal effects for genetic evaluation of egg production traits in dual-purpose chickens. Br Poult Sci.

[35] Liu J, Li M, Zhang Q, Xin W, Huang X (2020). Exploring the molecular basis of heterosis for plant breeding. J Integr Plant Biol.

[36] Su L, Wang SH, Han RL (2012). Polymorphisms of the PNPLA3 gene and their associations with chicken growth and carcass traits. Br Poult Sci.

[37] Zhang G, Fan Q, Zhang T (2015). Genome-wide association study of growth traits in the Jinghai Yellow chicken. Genet Mol Res.

[38] Amills M, Jimenez N, Villalba D (2003). Identification of three single nucleotide polymorphisms in the chicken insulin-like growth factor 1 and 2 genes and their associations with growth and feeding traits. Poult Sci.

[39] Faria DA, Peixoto JO, Lopes PS, Paiva SR, Silva PV, Guimarães SF (2009). Association between insulin-like growth factor I (IGF-I) microsatellite polymorphisms and important economic traits in pigs. R Bras Zootec.

